# A Review on the Production Methods and Applications of Graphene-Based Materials

**DOI:** 10.3390/nano11092414

**Published:** 2021-09-16

**Authors:** Md Abdullah Al Faruque, Md Syduzzaman, Joy Sarkar, Kadir Bilisik, Maryam Naebe

**Affiliations:** 1 Institute for Frontier Materials, Deakin University, Geelong, VIC 3216, Australia; m.alfaruque@deakin.edu.au; 2 Nano/Micro Fiber Preform Design and Composite Laboratory, Department of Textile Engineering, Faculty of Engineering, Erciyes University, Kayseri 38039, Turkey; s.zaman@butex.edu.bd (M.S.); kbilisik@erciyes.edu.tr (K.B.); 3 Department of Textile Engineering Management, Bangladesh University of Textiles, Dhaka 1208, Bangladesh; 4 Department of Textile Engineering, Khulna University of Engineering & Technology, Khulna 9203, Bangladesh; joy.sarkar@te.kuet.ac.bd

**Keywords:** graphene, graphene-based materials, graphene-based polymer composites, fibre-based reinforced nanocomposites, graphene applications, wearable technology

## Abstract

Graphene-based materials in the form of fibres, fabrics, films, and composite materials are the most widely investigated research domains because of their remarkable physicochemical and thermomechanical properties. In this era of scientific advancement, graphene has built the foundation of a new horizon of possibilities and received tremendous research focus in several application areas such as aerospace, energy, transportation, healthcare, agriculture, wastewater management, and wearable technology. Although graphene has been found to provide exceptional results in every application field, a massive proportion of research is still underway to configure required parameters to ensure the best possible outcomes from graphene-based materials. Until now, several review articles have been published to summarise the excellence of graphene and its derivatives, which focused mainly on a single application area of graphene. However, no single review is found to comprehensively study most used fabrication processes of graphene-based materials including their diversified and potential application areas. To address this genuine gap and ensure wider support for the upcoming research and investigations of this excellent material, this review aims to provide a snapshot of most used fabrication methods of graphene-based materials in the form of pure and composite fibres, graphene-based composite materials conjugated with polymers, and fibres. This study also provides a clear perspective of large-scale production feasibility and application areas of graphene-based materials in all forms.

## 1. Introduction

Since the discovery of graphene and its extraordinary electrical and mechanical potentials, graphene has been studied comprehensively and has become the centre of attraction for research and development [1,2,3,4]. Because of its amazing physicochemical and thermomechanical properties, graphene is thought to be the most promising aspirant for next generation materials. However, researchers are constantly focusing on this material to fine-tune its properties, upgrading its scalable production techniques, and potential application areas. So far, graphene has prospective for a wide variety of multifunctional applications, and accordingly, the graphene manufacturing industry has expanded dramatically. The graphene industry has shown vast scale development, but the flourishing of the graphene industry is yet to come, as the need for scalable production of graphene is a popular subject of research. Among the main manufacturing methods, mechanical exfoliation method [5,6], liquid phase stripping [7], oxidation-reduction method [8,9], and chemical vapor deposition [10] are most popular. The quality of graphene produced by these methods is exceptional, however, the cost of the preparation methods is a major constraint. Moreover, monolayer and high purity graphene are still a challenge, which limits the scalable production of graphene and its potential commercial applications [11].

**Figure 1:**
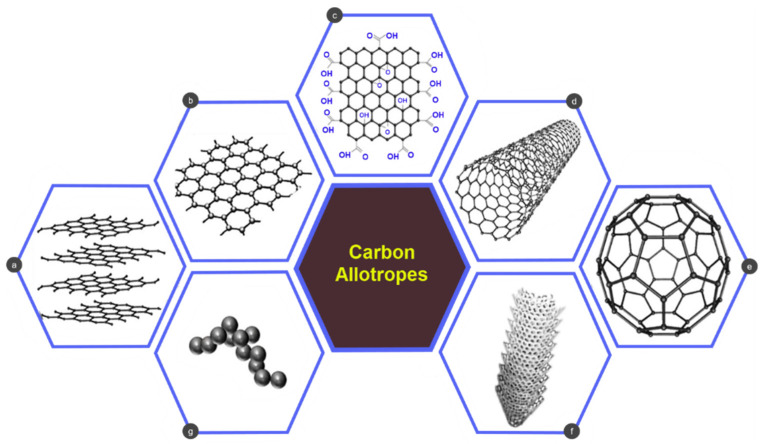
Carbon allotropic forms: (**a**) graphite, (**b**) graphene, (**c**) graphene oxide, (**d**) carbon nanotubes, (**e**) fullerene, (**f**) carbon nanofibers, (**g**) carbon dot; Reprinted with permission [28].

Graphene, the fundamental carbon allotrope, is formed by sp^2^-carbon atoms bonded together in a two-dimensional (2D) honeycomb lattice, and it can be synthesised either by top-down processes, e.g., mechanical/electrochemical/chemical exfoliation of graphite, or bottom-up methods, e.g., chemical vapor deposition and chemical synthesis [12,13]. As shown in Figure 1, 2D graphene is the fundamental element of all other carbon allotropes including 0-dimensional (0D) fullerenes, 1-dimensional (1D) carbon nanotubes (CNTs), and 3-dimensional (3D) graphite. All these allotropes can be obtained by modifying graphene [12,13,14]. In addition, graphene is light, solid, tough, demonstrating exceptional electrical and thermal conductivity, yet the thinnest and strongest among nanomaterials. Graphene is not only confined to the extreme level of thermal and electrical conductivity with fast electron mobility, and excellent mechanical strength, but its distinctive nanopore structure exhibits impermeability to gases, antimicrobial efficacy, thermal stability, excellent chemical resistance, high specific surface area, flexible surface chemistry, and eco-friendliness [15,16,17,18]. Among the various carbon allotropes, such as fullerenes, carbon nanotubes, graphene, graphene oxide, and carbon-based quantum dots, both the CNTs and graphene are the most studied materials due to their unique properties and are greatly used in wearable energy, batteries, biosensors, and in composites application areas [19]. Although CNT and graphene are almost alike in their properties, CNTs have some significant demerits in terms of toxicity and production cost [19,20]. It has been reported that if CNTs are worn for a prolonged time, they may trigger human body cell death, and cause oxidative stress, malignant transformation, destruction of DNA, inflammatory response, pulmonary inflammation, development of granulomas in lungs, scarring onto the skin and other organs [21,22]. Besides, formulation and synthesis of CNTs with other organic and inorganic chemicals are time-consuming processes [23,24]. Unlike CNTs, graphene is free from metallic impurities, which made it a more biocompatible material than that of CNTs [25]. Moreover, graphene can be synthesized from graphite, which is cheaper than the raw materials (carbon sources like methane) of CNTs [19,20]. Besides, graphene possesses a higher surface area than the single-walled carbon nanotubes (SWCNTs), which is advantageous for the electroactivity and immobilization capability. Moreover, for the presence of sp^2^ bonds in the structure and the electron configuration of graphene, it possesses an ultra-high mechanical strength, an electronic band that is tunable, outstanding thermal conductivity, and excellent elasticity [26]. As a result, though graphene was discovered later than the CNTs, it has attracted more interest compared to the CNTs [19,27].

Graphene-based materials have been used extensively in so many application areas such as in the field of energy, wearable technologies, agriculture, medical and healthcare, automobile, marine, and aerospace. The need for renewable energy to meet the pollution-related crisis of the current century can be addressed significantly with graphene/polymer composite materials. The solar cells especially, Li-ion batteries, and microbial fuel cells are prominent examples of the application of graphene/polymer composite in renewable and green energy. Additionally, human body temperature, blood pressure, and heartbeat rate can be easily monitored with sensors developed from graphene/polymer composite materials. Moreover, these materials can be used successfully in drug delivery systems, gene therapy, DNA sequencing, tissue engineering, and artificial bones, bioimaging, and potential cancer therapies [29,30,31,32,33,34,35]. The large surface area and flexible surface characteristics of graphene have particularly enhanced its application in wearable technologies [36,37,38]. The electrical properties along with the ability to be knitted and woven into cloth-like materials made graphene an attractive choice for wearable applications [39,40]. An exceptional example of the application of graphene in wearable technology is the utilisation of symmetrical and asymmetrical supercapacitors and micro-supercapacitors. In addition, the capacitance retention and cycle ability of the produced supercapacitors have been found very promising [41,42]. Graphene is considered the ultimate solution for clean and renewable energy materials and devices on a large scale. Low charging time, high strength to weight ratio, and large surface area of graphene made it a strong candidate for use in batteries and cells with remarkable performance [43,44,45].

Until now, several published review articles have explored the production process, physical and chemical properties, as well as applications of graphene-based materials, which can be used in diversified application areas [46,47,48,49,50,51,52]. However, almost all primarily focused on a single application field and studied the material development, their characteristics, and applications only for that particular area, for example, sensors and biosensors [53,54,55,56], energy storage and supercapacitors [57,58,59,60,61,62], and biomedical and drug delivery [63,64,65,66]. There is a scarcity of review articles on graphene-based materials to comprehensively cover all the production processes of graphene derivatives such as pure and composite graphene fibres, graphene/polymer composite (gPCs), and graphene/fibre/polymer composites (gFPCs), and their applications in various fields such as energy, wearable technology, agriculture, wastewater treatment, medical, healthcare, and in the automobile industry. Therefore, this review aims to highlight the broadest views of all the possible applications of graphene-based composite materials along with their detailed fabrication methods. As graphene has the potential to address the global concerns related to energy and pollution, it is important to know the state of the art, specific properties, and potential application areas of graphene-based materials.

## 2. Production Process of Graphene-Based Materials

The production and use of graphene-based materials are continuously increasing over the last two decades. The number of articles published from 2001 to 2020 on the graphene-based composite materials (such as fibres, fabrics, films, polymers composites, and fibre polymer composites) and their different production methods are summarised in Table 1 and Table 2, respectively.

It is evident that the research interest on the graphene-based materials and their manufacturing techniques is increasing day-by-day. The following describes the most used approaches of the production of graphene-based materials.

### 2.1. Spinning of Graphene Fibres

Graphene fibres can be fabricated using solution spinning techniques such as wet spinning, dry spinning, and dry jet spinning processes. The fabrication of pure graphene fibres using the melt spinning technique is not applicable because of its high temperature at melting point (~4600 °C) [67]. Therefore, the solution spinning technique is the primary technique, and among all, wet spinning is extensively used in the fabrication of pure graphene fibres and graphene-based composite fibres. After wet spinning, the graphene fibres pass through reduction processes such as physical, chemical, and thermal reduction to achieve electrical conductivity [8,9,68,69,70].

**Figure 2:**
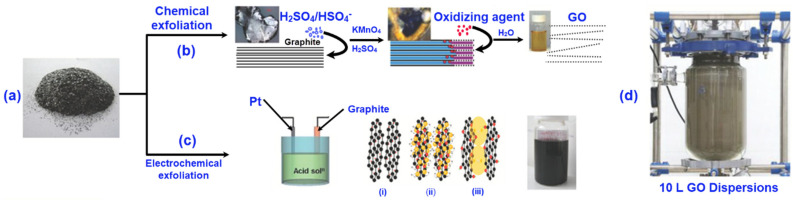
Chemical and electrochemical exfoliation of graphene oxide (GO) from graphite. (**a**) graphite, (**b**) chemical exfoliation approach, (**c**) electrochemical exfoliation approach, and (**d**) GO dispersion in water with a volume of 10 L; Reprinted with permission from [50].

Before the fabrication of the pure graphene fibres, graphite is required to be converted into graphene oxide (GO). This is because the 2D structure of graphite is made of weaker intermolecular bonding, difficult to reform into a fixed attachment by the direct layer assembling [71]. Besides, it is tough to make a uniform solution where the graphene flakes can be dissolved uniformly to regenerate the fibres. Moreover, it is troublesome to organize the graphene flakes in a uniform order next to the fibre axis to produce the graphene fibres [72]. Therefore, to overcome these difficulties graphite is transformed into GO that contains an ample amount of oxygen functional groups, which can easily be dissolved into water or other polar organic solvents [73,74]. To produce GO from the graphite on a large scale, the chemical and electrochemical exfoliation processes follow as shown in Figure 2 [75,76]. In the case of the chemical exfoliation approach, both the Hummers and modified Hummers methods are used to convert the graphite (Figure 2a) into GO, in which potassium permanganate, sulfuric acid, and hydrogen peroxide play an important role in the delamination and oxidisation of the graphite sheets [50,77]. The modified Hummers method is briefly discussed (Figure 2b); the expanded graphite (1 g) and sulphuric acid (H_2_SO_4_, 200 mL) are mixed and stirred continuously overnight. Then potassium permanganate (KMnO_4_, 10 g) is added very slowly until the colour of the mixture turns green. Next, de-ionised water (DI, 200 mL) is gradually added, changing the colour of the mixture from green to purple to brown. After that, hydrogen peroxide (H_2_O_2_, 30 mL) solution is added dropwise into the mixture until the colour of the solution turns light yellow. Subsequently, hydrogen chloride (HCl, 500 mL) solution (9:1 *v*/*v*, water to HCl) is added into the mixture and stirred for 30–40 min before it is centrifuged for 20~30 min. Finally, the solution is repeatedly washed around 6–7 times with DI water [78,79]. In the electrochemical exfoliation approach (Figure 2c), an electrical field is introduced to the graphite ultimately infusing the electrons, making graphite positively or negatively interpolated. Finally, during solvent electrolysis in the gas expansion process, the graphite is exfoliated into GO sheets [50,77]. These GO sheets are later used to produce pure GO fibres via wet spinning.

**Figure 3:**
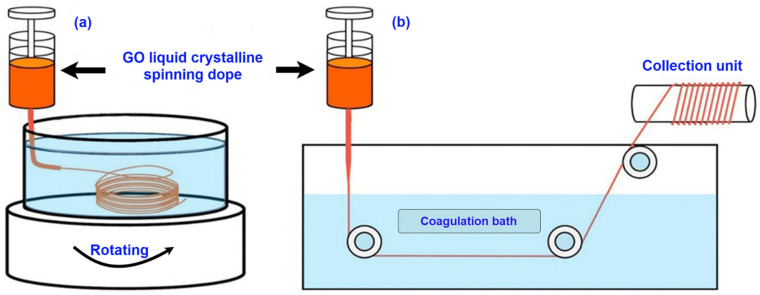
(**a**) Wet-spinning process of GO fibres, (**b**) collection process after the coagulation process; Reprinted with permission from [67].

The wet spinning of graphene fibres has been investigated widely because it is an easy process, lower prerequisite of apparatus, and homogeneous dispersibility of GO sheets in the solvents [80]. To produce the graphene fibres via wet spinning, a dope solution is prepared where GO is dissolved in the suitable solvent and then passed through the coagulation bath. The coagulation bath can be prepared with a mixture of solvent and non-solvent, where the dope solution is converted into a gel-like structure. Finally, after the drying process, this gel-like structure is transformed into graphene fibres [80]. This technique is a combination of a double-diffusion, where the solvent present in the dope solution can coagulate inside the coagulation bath, and the coagulating agent can be inserted into the fibres [81,82,83]. When the dope solution is injected into the coagulation bath, to confirm the formation of consistent and uninterrupted GO fibre, the bath rotates continuously (Figure 3a) or the fibres are collected after passing through the bath to the collection unit at a uniform draw ratio (Figure 3b) [67]. Although it has been reported that in the first approach (Figure 3a), GO fibres can be fabricated with an extraordinary tensile strength (between 185 MPa and 365 MPa), it is not suitable for the large-scale production of GO fibre, as the rotating speed of the coagulation bath and the fibres requires precise control [50,67,84]. It is more convenient to produce GO fibres with a constant drawing and collecting onto a collection unit, after passing through the coagulation bath. This approach (Figure 3b) is also more feasible for producing GO fibres on a large commercial scale [85].

The preparation of a suitable dope solution is an important step to assure the optimum spinnability of the GO fibres. Water is mostly used to disperse or dissolve the GO sheet in the production of the GO dope solution. The size of the GO sheets, viscosity, and the concentration of the GO dope solution are also important factors affecting the spinnability of the GO fibres [86]. It has been found that the larger GO sheets are difficult to dissolve properly in the solvent, while highly concentrated and viscous GO dope solution is difficult to pass through the nozzle that eventually limits the spinnability of the GO fibres [87,88,89]. Filtration, centrifugation, and deaeration of the dope solution are some of the processes that enhance the quality of the GO dope solution [50]. In recent years, the formation of liquid crystalline graphene oxide (LCGO) dope solution has been used to fabricate the GO fibres, where both the aspect ratio and solubility of LCGO are the key elements that ascertain the articulation of the LCGO [50,71,86].

**Figure 4:**
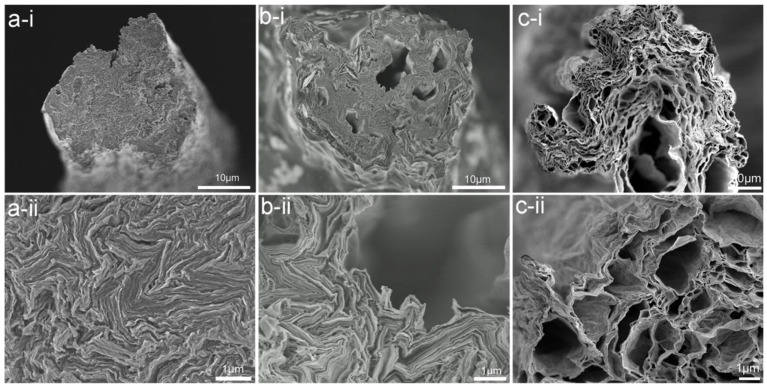
The morphological structure of the graphene fibres at different magnifications, produced from various coagulation bath: (**a**-**i**) and (**a**-**ii**) water, (**b**-**i**) and (**b**-**ii**) acetone, and (**c**-**i**) and (**c**-**ii**) acidic medium (pH-3); Reprinted with permission from [78].

Xu and Gao discussed the properties of the LCGO solution by preparing an extensively soluble single-layered GO with a high width/thickness ratio (aspect ratio) [90]. Later, the authors demonstrated the continuous production of the wet spun GO fibres from the LCGO using the wet-spinning technique [91]. The utilisation of a proper coagulation bath for the precipitation and solidification of the GO dope solution is also an important sphere of consideration while performing the wet spinning of the GO fibres [50,92,93]. In general, a mixture of water and alcohol, in different volume ratios is extensively used as a coagulating agent [50,86]. In addition, the use of KOH, NaOH, CuCl_2_, CuSO_4_, and CaCl_2_ is reported as a precipitating agent, where the presence of excess metal ions can be removed by the subsequent washing, drying, and thermal treatment of the fibres [86,94,95]. Figure 4 shows the morphological structure of the graphene fibres produced with different coagulation baths (water, acetone, and acidic medium, from a to c, respectively) [78]. As it is observed with the change of the coagulant in the coagulation bath, the morphology of the fibres changes from the non-porous and dense structure to the porous and loosely packed fibres [78].

**Figure 5:**
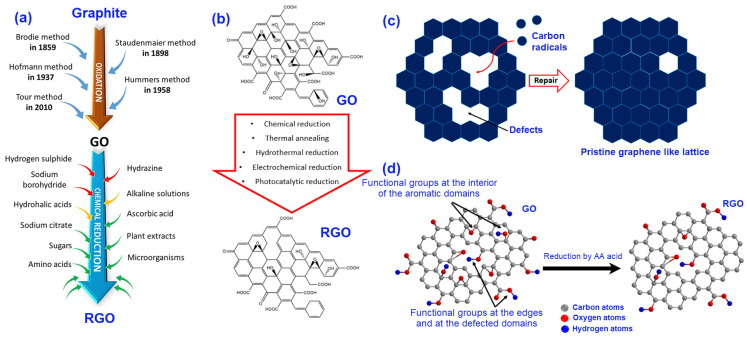
(**a**) Schematic diagram of fabricating RGO from graphite [97], (**b**) Steps followed for the conversion of GO into RGO [68], (**c**) Schematic diagram of repairing the defects of the pristine graphene [68], and (**d**) Changing of the oxygen functional groups after reduction using ascorbic acid [98]. All the figures were reprinted with permission.

After the spinning process, the dried graphene fibres go through the chemical reduction processes (chemical reagents, photocatalysis, and electrochemical reduction), or the thermal reduction process (thermal annealing, and microwave and photo reduction). The reduction process aims to remove the oxygen-containing groups from the fibres and repair the lattice defects of the graphene structure (graphitic network), which will eventually increase the electrical conductivity and other functional properties of the fibres [8,67,86,96]. Different chemicals such as hydrazine and its derivatives, hydroiodic acid (HI), and metal hydrides (e.g., sodium hydride and sodium borohydride) are used to reduce the GO fibres [8,9]. Since some of these chemicals are hazardous, corrosive, and toxic, more recently eco-friendly and green chemicals including organic acids, microorganisms, plant extracts, antioxidants, and sugars are used to accomplish the reduction of graphene fibres [97]. Ascorbic acid (AA, also known as Vitamin C), Caffeic acid (CA), a mixture of ascorbic acid, and sodium-citrate are some of the examples of excellent green reducing agents of GO fibres [8,98]. It has been reported that the chemical reduction approach of the GO fibres is highly suitable and industrially scalable compared to the thermal reduction process. This step is accomplished at room temperature (or slightly higher up to 90 °C), it is simple, cheap, and importantly does not require large instruments for the reduction process set-up [68,96,99]. Figure 5 demonstrates different approaches of fabrication to reduce GO (RGO) from graphite, along with the steps using the reduction of GO, schematic diagrams of the removal of the defects, and changing of the oxygen functional groups after reduction.

### 2.2. Graphene Polymer Composites (gPCs)

**Figure 6:**
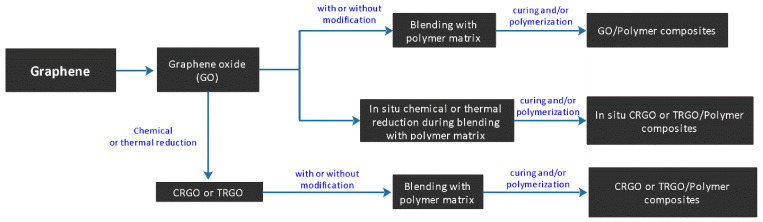
The general fabrication routes for polymer-based composites with GO or RGO as fillers; Reprinted with permission from [101].

The most commonly used techniques of gPCs fabrications are; solution mixing, melt blending, in situ polymerization, and high shear mixing–calendaring, which are discussed in detail in the following sections [100]. General fabrication steps for graphene/polymer composites are delineated in Figure 6 [101].

It is to be noted that the composite fabrication techniques influence the dispersion of graphene and/or its derivatives in the polymer matrices and will affect the performances of the composite materials [80]. The molecular weight, polarity, hydrophobicity, and reactive groups of the polymer resin, graphene nano-fillers, and solvent are considered the main controlling parameters in graphene/polymer composite synthesis [102,103]. Pristine graphene cannot be evenly dispersed in most aqueous solvents, because of its hydrophobic nature. Therefore, even though graphene derivatives such as graphene-oxide (GO), chemically reduced graphene oxide (CRGO), and thermally reduced graphene-oxide (TRGO) have lower physical properties than pristine graphene, they are being used as fillers when making composites. The hydrophilic modified graphene is preferred to boost industrial production and the applications of polymer composites [104].

#### 2.2.1. Solution Mixing

**Figure 7:**
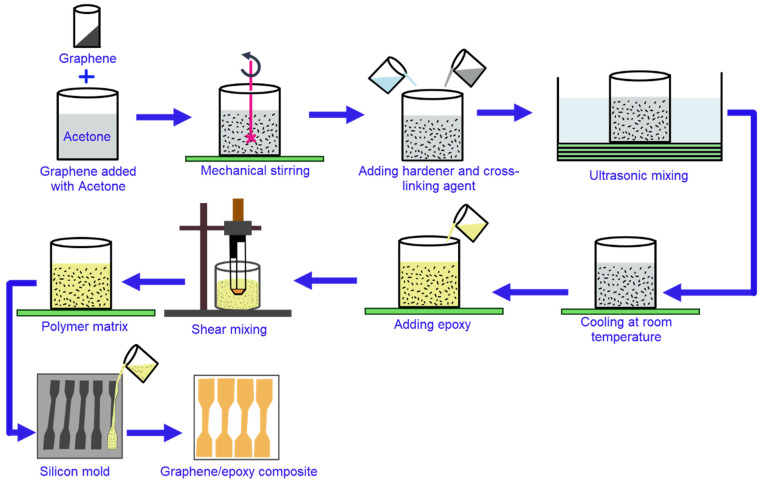
Step-by-step process flows of graphene/polymer nanocomposite manufacturing.

Solution mixing is the easiest and the most widely used technique for the large-scale production of graphene/polymer composites. This method is suitable for both thermoplastic and thermoset polymer resins. It usually involves three steps including (i) Dispersion of filler in a suitable solvent, (ii) Incorporation of the polymer, and (iii) Removal of the solvent by distillation or evaporation [105,106]. As shown in Figure 7, the graphene or graphene derivatives are firstly dispersed in a suitable solvent like water, acetone, chloroform, tetrahydrofuran (THF), dimethylformamide (DMF), or toluene followed by sonication, mechanical or magnetic stirring. Then, this graphene suspension is mixed with the polymer resin either in the same solvent or in a mixed solvent via the shear mixing or stirring process. Finally, the solvent is evaporated, and the newly formed nanocomposite is washed with distilled water to remove the remaining solvents followed by drying to obtain the graphene/polymer composites. Although the fabrication of uniform and homogenous dispersion of graphene nanomaterials is possible with this method, solvent removal is a critical issue [105,107]. The selected solvent must be compatible with the polymer resin and be volatile to facilitate the evaporation or distillation processes [105,108]. A wide range of polymers including epoxy [109], polyvinyl alcohol (PVA) [110], polyvinyl fluoride (PVF) [111,112], polyethylene (PE) [113,114], polypropylene [115,116], polymethylmethacrylate (PMMA) [117,118], polyurethane (PU) [119,120], polystyrene (PS) [121,122] have been explored and found to be suitable for graphene/polymer composite manufacturing via the solution mixing technique. Consequently, polymer resin can intercalate between the graphite layers more easily during the composite fabrication process, thus, resulting in a uniform distribution of graphene or modified graphene materials in the polymer resin [123]. Though this is the simple technique to make graphene/polymer composite, it is, however, challenging to completely remove the organic solvents as well as the air bubbles trapped inside the structures, thus, causing deterioration in the structural and functional properties [124].

#### 2.2.2. Melt Blending

Melt blending is another practical and popular composite fabrication technique, especially for thermoplastic polymer composites. It is eco-friendly, cost-effective, and also worthy of large-scale manufacturing of nanocomposites [125]. This technique requires no solvent for processing and the graphene or modified graphene materials are integrated with the molten polymer matrix. Solid graphene materials at a higher temperature are mechanically mixed with the molten thermoplastic polymers in a twin-screw extruder. The high shear force in the twin-screw extruder pushes the graphene materials to intercalate inside the polymer structures. The uniform dispersion of graphene materials depends on the extent of polymer disintegration at higher temperatures. The properties of the resultant polymer nanocomposites can be regulated by controlling various fabrication process parameters including rotation speed of the screw, mixing temperature, and time [124]. A wide range of polymers including PU [126], PET [114], polylactic acid (PLA) [127], isotactic polypropylene (iPP) [128], styrene, and acrylonitrile [129], polyamide (PA) [129,130] and polycarbonate [131], have been explored so far and found to be suitable for graphene/polymer composite manufacturing via melt blending technique. Although this technique is an environmentally friendly method as no toxic solvent is used, it has some significant drawbacks such as heterogeneous dispersion of graphene, which might be due to the high viscosity of graphene–polymer dispersion even at a lower loading of graphene [132]. In addition, defects and breakage of graphene sheets like buckling, rolling, and even shortening due to the higher shear forces used during mixing in the twin-screw extruder, and poor conductive properties of the composite due to the reduced aspect ratio of graphene sheets, are also some of the disadvantages of this method [101].

#### 2.2.3. In Situ Polymerisation

In-situ polymerisation is an effective technique ensuring the homogenous dispersion of nano-fillers in the polymer matrices. This technique facilitates the formation of a strong interaction between the polymer matrix and reinforcement (e.g., rGO). In this technique, graphene or modified graphene is first mixed and swollen in a monomer solution. Then, these graphene materials are ultrasonically dispersed in the solution and a suitable initiator is added. Finally, the polymerisation process commences either by heat or radiation [104]. The viscosity of the graphene–polymer mixture is dependent on controlling the degree of polymerisation. This is because the viscosity of the mixture increases with the reaction. Finally, the composite structures are obtained either by following the precipitation/extraction or solution casting process [100]. A large number of graphene/polymer nanocomposites including PU [133], PS [134], PMMA [135], polyimide (PI) [136], and PET [114] have been prepared by this method. One of the notable advantages of this technique is the possibility of a high level of homogenous graphene dispersion in the polymer matrix. Moreover, it facilitates a strong covalent bonding between the polymer and graphene materials. However, there are some challenges to be considered in this technique, such as the increased graphene–polymer mixture viscosity with a higher degree of polymerization, which ultimately attributes to poor manipulation and inferior load fraction in the composite structures [108].

#### 2.2.4. Roll to Roll Milling

Roll to roll (calendaring) milling is also another technique that ensures the homogenous dispersion as well as the high filler contents of polymer matrices to improve composite performances [137,138,139,140]. This technique is suitable particularly for thermoset polymer resins such as epoxy resins. The required amount of graphene and polymer resin are placed between the (usually two) rotating rollers and uniformly mixed under a high shear force by reducing the roller gaps [139,141]. Milling time and shear forces controlled by changing the gap between the rollers affecting the homogenous dispersion of graphene and/or some other nanomaterials in the polymer matrices. This technique seems quite feasible for industrial fabrication, though batch-to-batch quality variation may occur depending on the feeding accuracy and process control [105,142]. However, this technique is labour intensive and often difficult to automate.

### 2.3. Graphene/Fibre/Polymer Composites (gFPCs)

The graphene nanomaterial can be incorporated with the fibre-reinforced polymer composites (FRPCs) by following three methods as described below. In the first method, referred to as the ‘Matrix Modification Method’, graphene is mixed with a suitable polymer and then applied to the reinforcement fibre by either dip coating, hand lay-up, or spray-up techniques. In the second method, known as the ‘Fibre Modification Method’, graphene nanomaterial is directly integrated onto the fibre surfaces via electrophoretic deposition (EPD), chemical vapour deposition (CVD), and chemical grafting techniques. The third method is the combination of the two methods, where graphene can be incorporated with both the fibre and polymer matrix simultaneously to boost the composite properties for the desired end uses [100].

#### 2.3.1. Matrix Modification Method

**Figure 8:**
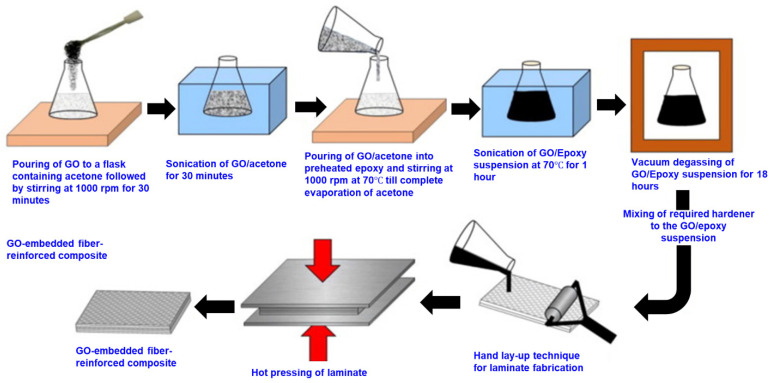
Fabrication steps of the gFPCs via matrix modification method; Reprinted with permission from [156].

This is the most widely used method to introduce nanomaterials with the polymer composites. The fabrication steps of the gFPCs via the matrix modification method are demonstrated in Figure 8. A certain amount of graphene (wt.%) is mixed with the polymer resin by a wide variety of mixing techniques including shear mixing either mechanical or magnetic [143,144], extrusion [145], rolling (calendaring), or ball milling [146], and ultrasonication [147]. These techniques can also be applied together to ensure the proper dispersion of the nanomaterials resulting in the ultimate desired properties of the composite structures [148]. After mixing the graphene nanomaterial with polymer resin, a selective hardener and an accelerator are added followed by the degassing step. The polymer matrix is pumped out to reduce the void contents in the matrix. The physicochemical properties of the polymer matrix differ based on the processing conditions, the type, and amount of chemicals used with the resin [149]. Graphene can be incorporated with the reinforcement textile fibre or fabrics either via dip coating of the fibre into the polymer matrix [150] or hand brushing [151] or spraying techniques [152]. After that, graphene incorporated textile preforms are pre-cured at a higher temperature (approximately around 110 °C) for a certain time (5–10 min) depending on the type of polymer matrix and reinforcement fibres to obtain the prepregs. These prepregs are stacked in a particular sequence and placed in a particular mould. Finally, the nano prepreg structures are hot press cured under a certain pressure (e.g., 0.6 MPa) at a certain high temperature (e.g., 150 °C) for a period of time (e.g., 15 min) [153]. Note that curing conditions, including pressure, temperature, and pressing duration, are found to tailor the composite properties. Nevertheless, the non-uniform dispersion and agglomeration of graphene in the matrices create some drawbacks such as lower wettability and poor interfacial adhesion between the fibres and matrix, which lower their mechanical properties and restrict the wider applications [154,155].

#### 2.3.2. Fibre Modification Method

Graphene nanomaterials can be directly incorporated with the textile preforms including fibre, tow, and fabrics. It has been found that electrophoretic deposition (EPD) and chemical vapour deposition (CVD) methods are the most commonly used techniques to graft graphene and some other nanomaterials including amorphous carbon, carbon nano-fibres, and carbon nanotubes [157].

##### Electrophoretic Deposition Technique (EPD)

**Figure 9:**
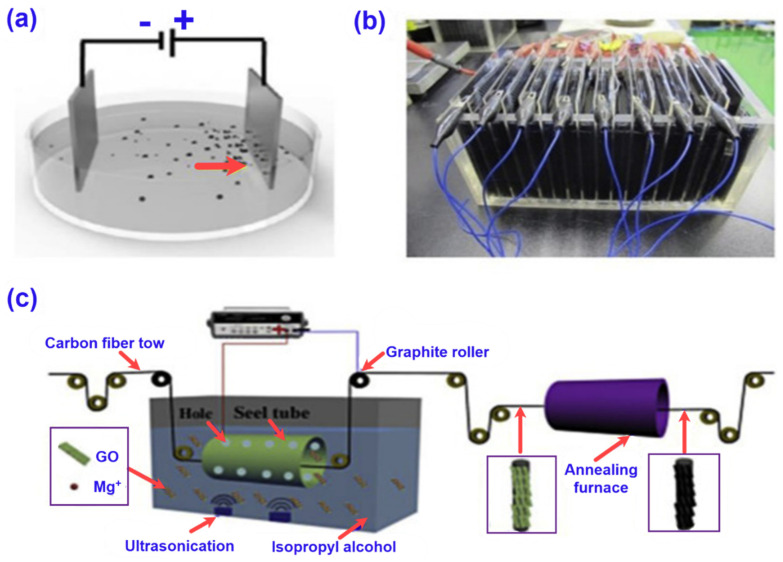
The schematic of depositing graphene or modified graphene on the reinforcement fibres by the EPD technique. (**a**) Typical EPD equipment for the deposition of GO with positive and negative electrodes aligned in parallel [170], (**b**) EPD equipment that can produce 16 pieces of GO/CNT coated carbon fabrics simultaneously [171], (**c**) Equipment of continuous EPD of graphene on carbon fibres [172]. All the figures were reprinted with permission.

Electrophoretic deposition (EPD) is a low-cost and practical technique to introduce the graphene nanomaterials onto the surface of the textile preforms [158,159]. The active surface area of fibre is enhanced to improve the interfacial adhesion properties between the textile preforms and polymer matrix [160]. Since pristine graphene is hydrophobic and uniform dispersion in aqueous solvents is not obtained, oxidised graphene is preferred to pristine graphene while making the EPD solution. The negatively charged GO, high mobility, and hydrophilic oxygen functional groups on GO make it suitable for EPD solution [161]. Figure 9 demonstrates the schematic of the EPD technique. In this method, textile preforms are mounted onto the positive electrode, and negatively charged GO moves towards the preforms when an electrical voltage is applied to the electrodes immersed in the solution. In this way, GO is deposited on the textile preforms [162]. Stability and concentration of GO in the suspension [163], the size of the suspended graphene, applied voltage [164,165], and deposition time [166] are the most influencing factors for graphene uniform deposition. Sometimes, surfactants are used to keep the suspension stable by providing a higher zeta potential and electrophoretic mobility to the suspended particles in the solution. However, there are some drawbacks in EPD including bubbles on electrodes caused by the electrolysis process and preform damage and micro-cracks generated due to the impurities deposited. The cracks are found to propagate quickly under loads leading to structural failure [167]. To reduce the bubbling effect in the dispersion solution, sonication is applied and it consequently enhances the quality as well as quantity of the graphene deposition [168]. If the EPD parameters can be controlled properly, it is an excellent technique for the homogenous graphene coating on the fibre surface. This technique can be easily used on an industrial scale by adjusting the treatment conditions [169].

##### Chemical Vapour Deposition Technique (CVD)

**Figure 10:**
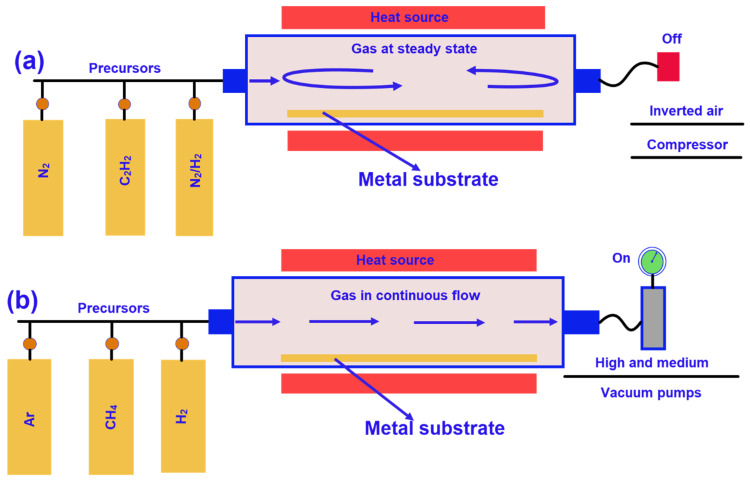
Schematic representation of the chemical vapour deposition of graphene synthesis (**a**) at atmosphere pressure using polycrystalline substrate and gas discontinuous flow, (**b**) at high vacuum using monocrystalline substrate and gas continuous flow; Reprinted with permission from [190].

The chemical vapour deposition technique has recently received considerable attention because of the large-scale graphene synthesis with controlled architecture and low defects [173,174,175,176]. The CVD technique encompasses four main stages: (i) activation of feed gases, (ii) chemical reaction, (iii) formation of graphene nanomaterials, and (iv) deposition on a suitable substrate. Graphene can be deposited either on a temporary metal substrate such as Fe [177], Cu and Ni [178], Pt [179], or their alloys and then transferred onto the textile preforms or directly deposited on the textile preforms. The carbon-containing gases (e.g., hydrocarbon gases) are used as the precursors in the CVD technique. The feed precursors are pyrolyzed at elevated temperature to form different carbon allotropes including graphene, carbon nanotubes, and amorphous carbon. Furthermore, a variety of metal catalysts like Co(NO_3_)_2_.6H_2_O, Fe(NO_3_)_3_.9H_2_O, and Ni(NO_3_)_2_·6H_2_O are used to reduce the temperature of the CVD process and to boost the pyrolysis of the feed gases. These catalysts act as the active sites for depositing graphene film. The annealing process at the elevated temperature causes H_2_ to dissociate into atomic H, which ultimately leads to the dehydrogenation of hydrocarbon precursors and carbon radicals start to deposit onto the metallic substrate integrated with high-performance fibres [180,181]. Graphene growth rate predominantly relies on the hydrogen diffusivity and solubility of these catalysts [180]. The CVD furnace temperature, reaction duration, feed gases, feed ratio, catalysts, and their molar ratios are the crucial factors for the type and quality of deposited nanomaterials. Temperature is one of the most influential factors in graphene production via the CVD technique. Graphene growth rate increases as the furnace temperature increases and more importantly, better quality monolayer graphene film with minimum defects is produced in a higher temperature [182,183,184]. The production rate of graphene also varies due to pressure differences in the furnace. For example, single layer graphene is usually produced at lower pressure whereas the bi-layer and multilayer graphene films are produced at higher pressure (more than 50 mbar) [185]. Hydrocarbon precursor is another significant factor in the CVD technique. Precursor (e.g., CH_4_) concentration plays an important role in the graphene film growing kinetics. Monolayer graphene and multilayer graphene film are found to be deposited onto the metal substrate while using the lower and higher concentration precursors, respectively [186]. Acetylene, another kind of most commonly used precursor, is better than methane because of its better pyrolysis performance. The lower feeding rate of C_2_H_2_ results in less defective graphene [187]. Although the CVD technique is commonly used for the large-scale production, this process is currently highly time-consuming, costly, and complex. It is also difficult to control the thickness of graphene deposited on the fibre surface, and, most importantly, because of using higher temperature, the mechanical properties of the fibres are deteriorated [188,189]. The CVD technique of graphene synthesis is schematically shown in Figure 10.

#### 2.3.3. Chemical Grafting Technique

**Figure 11:**
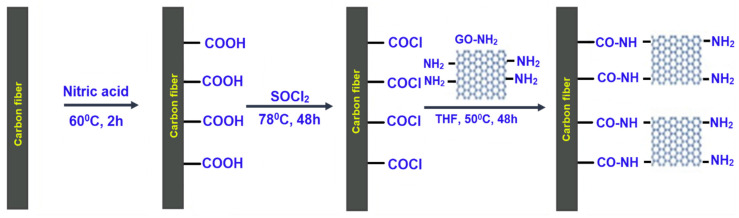
Schematic of grafting the functionalised graphene using SOCl_2_ as the connecting agent; Reprinted with permission from [194].

This is one of the latest techniques to graft graphene or its derivatives onto the surface of the fibres. Chemical grafting is a way of connecting graphene with high-performance fibres such as carbon and aramid fibres by the covalent bonding via a variety of connecting or coupling agents such as hexamethylene diisocyanate (HDI) tri-polymer, poly(amidoamine) (PAMAM) dendrimers [191,192,193]. Functionalised graphene has been directly grafted onto the surface of carbon fibre using a newly developed method [194,195,196], where SOCl_2_ is used as the connecting agent (Figure 11). In this technique, firstly, the carbon fibres are oxidised by treating with concentrated nitric acid (HNO_3_) and functional carboxylic groups (-COOH) are introduced onto the fibre surface. Then, a thin layer of the coupling agent is coated on the carbon fibre surface and finally is treated with modified graphene to be grafted onto the fibre surface. This technique involves some demerits; it is costly, requires multisteps for grafting, uses toxic and corrosive chemicals, damages the fibre surface and deteriorates mechanical properties of fibres, and sometimes it is not possible to remove the chemicals from the fibre surface completely [191,197].

## 3. Applications of Graphene-Based Materials

Graphene-based materials are found in a wide variety of advanced applications because of their extraordinary structural and functional properties [198]. As a result of intense research on graphene over the past decades, it is now being used in so many application fields ranging from agriculture to aerospace. Some of the most remarkable uses of graphene-based materials include solar cells, supercapacitors, Li-ion batteries, microbial fuel cells, sensors, and nanomembranes for wastewater treatment. In addition, graphene nanomaterials are being used for various important medical purposes such as drug delivery systems, gene therapy, DNA sequencing, tissue engineering and artificial bones, bio-imaging, and potential cancer therapies. Moreover, graphene incorporated fibre-reinforced composites are noticeably occupying the automotive, marine, aerospace industry due to their high strength to weight ratio properties.

### 3.1. Energy

**Figure 12:**
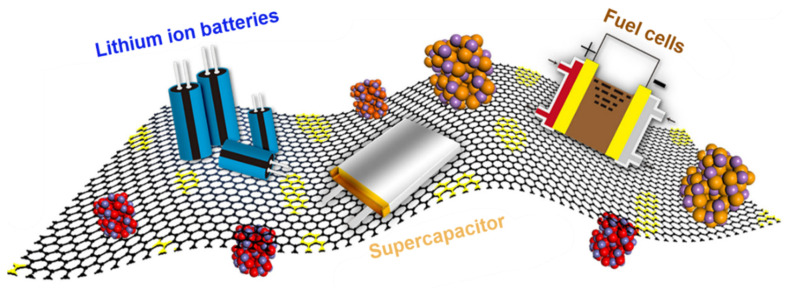
Applications of graphene-based materials in energy storage and energy conversion devices; Reprinted with permission from [209].

Graphene-based materials are used in producing renewable energy, which is one of the critical issues of today’s world. Different types of solar cells are produced from graphene to convert solar energy into other forms of energy [199]. For instance, graphene-based solar cells are used for solar-thermal or solar-electrical conversion, and photo-catalysis applications [200]. Among some remarkable properties of graphene such as the 2D structure, excellent electrical and thermal conductivity, high transparency, flexibility, exceptional mechanical strength, and very large specific surface area, the enhanced electrical conductivity without affecting the optical transmittance is the best reason for its success in the solar cells. Moreover, the hydrophobic nature of graphene is thought to prevent unwanted reactions from impeding degradation [201]. In addition, graphene/polymer composites are now used as the electrodes in the dye-sensitised solar cells (DSSC) to convert solar energy into electrical energy. The energy conversion efficiency has been substantially improved using this type of electrodes because of their larger surface area, high porosity, better conductivity, and reasonably good chemical stability [202,203]. Graphene-based conducting polymer composites are used in supercapacitor or ultracapacitor and have been found to demonstrate improved performance as compared to conventional batteries [204]. It is possible to fabricate an ultrathin (<100 nm) graphene film by filtration method, where this film can be transformed onto a flexible substrate, such as polyethylene terephthalate (PET). That is why graphene is a strong candidate to be used in the supercapacitors’ electrode intended for flexible and wearable energy storage devices [205]. Furthermore, if graphene is compounded with polymer binder, it can compensate the limitations of insulating polymers. On the other hand, it enhances the mechanical properties of the polymer framework, resulting in a praiseworthy improvement in cycling ability and specific capacitance of conductive polymers [206]. Polyaniline (PANI) is one of the most used conducting polymers with graphene oxide or reduced graphene oxide to make high-performance capacitors [207,208]. These graphene-based capacitors provide high power density, short charge and discharge time, and long-life cycle of the capacitor. Figure 12 represents the applications of graphene-based materials in energy storage and energy conversion devices [209].

Li-ion battery (LiB) is another prominently used storage system. However, the anode and cathode materials show low life due to the continuous volume expansion and contraction caused by the lithiation/de-lithiation reaction [210,211]. Furthermore, Li-ion batteries fail to perform as expected due to the low electron conductivity properties of the existing electrodes. The problem has been solved using graphene-based anode and cathodes in the Li-batteries. Li-ion batteries demonstrated excellent performance because graphene-based electrodes possess good electrical conductivity and reasonably high porous structures [212,213,214]. The fabrication methods and properties of graphene-based materials used in energy storage and conversion devices are provided in Table 3. Graphene/polymer composites are also found to be used in microbial fuel cells to produce electricity from different organic sources [215].

### 3.2. Wearable Technology

Graphene-based materials are the subject of huge attraction in the field of wearable technologies. The aim is to assemble fibre-based supercapacitors that are flexible with excellent electrochemical properties. For example, supercapacitors (SCs) are one of the popular examples of wearable storage and conversion devices. SCs are being used in hybrid electric vehicles, energy management, memory backup devices, and industrial power [220]. Different approaches have been taken to use graphene-based fibres to devise supercapacitors for energy storage and wearable technologies [39,220,221,222,223,224,225,226,227,228,229,230,231,232]. Among these, an asymmetric supercapacitor with high flexibility, excellent cycling ability, and mechanical stability is remarkable. Moreover, its superior volumetric energy density made it reversibly cycled at a high voltage of 1.6 V [223]. Transition metal oxide nanorods/reduced graphene oxide hybrid fibres were used for the purpose. The electrochemical performance of the hybrid fibres considerably increased as a result of the synergetic effects between transition metal oxide nanorods and rGO [223]. Whereas in an all-solid-state symmetric supercapacitor, due to the improved electron transportation of the conductive graphene network, significant electrochemical properties were also found even under bending and stretching conditions, which indicates the suitability of the material to be used in wearable technologies [224]. Other supercapacitors and micro-supercapacitors are mentioned as strong candidates for wearable technologies. Among the various composite fibres used in SCs, a specific capacitance of 1722.1 mF cm^−2^ and energy density of 37.2 µWh cm^−2^ have been shown by the graphene fibre fabricated with a titanium core that was prepared using the alternately dipping (AD) technique [226]. In another study, the fabricated graphene and MnO_2_ hybrid supercapacitor demonstrated up to 93% capacitance retention after 1000 cycles, which was a promising outcome to apply in the field of wearable fabric [228]. Polyurethane yarn as the elastic core and graphene/poly (vinyl alcohol) as the conductive sheath fabricated using layer by layer assembly method can be used as a yarn strain sensor for their superior electromechanical properties and good linearity between the change in relative resistance and applied strain [233]. In every case, the graphene’s excellent conductivity and remarkable mechanical properties with flexible structure assisted the fabricated devices to perform assertively. Dopamine-modified aramid fibres (AF) and Graphene/TiO_2_ continuous fibres (GTF) are prominent candidates for use in environmental remediation [218,219]. Moreover, Graphene/TiO_2_ continuous fibres (GTF) have photocatalytic activities under both UV irradiation and visible light irradiation [218].

**Figure 13:**
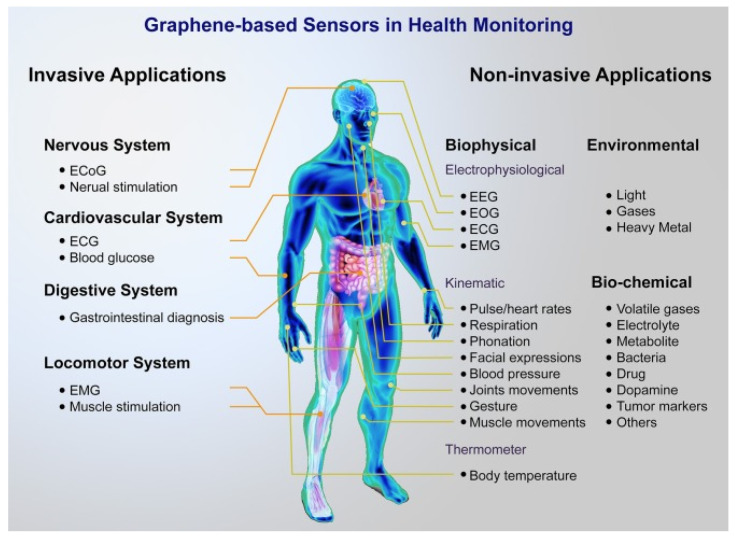
Brief of a graphene-based sensor platform for health monitoring; Reprinted with permission from [245].

Graphene quartz fibre (GQF) are suitable for industrial electronic heaters and real-time biomimetic gas sensor [234,235]. Gas sensors are used to screen the air quality, environment, and to sense the presence of different toxic gases such as carbon dioxide, methanol, ethanol, ethylene, formaldehyde, and acetone [236]. The performance of the gas sensors are determined using some parameters, for example, selectivity, sensitivity, response time, detection limit, and response recovery [236,237]. The preparation process of a graphene-based gas sensor is easier and more cost-effective than the other metal-based sensors, which eventually increase its applications in diversified areas [236,237]. They can be knitted into meter-scaled knit fabrics with excellent electrothermal conversion efficiency, high sensitivity, fast response (<0.5 s), and good durability (~5000 cycles) to organic solvent vapour [234]. As shown in Figure 13, graphene/polymer composites are used in different sensor elements, especially for healthcare [238] and environmental control systems [239]. The graphene coated or incorporated fibre or fabric-based gas sensors possess excellent advantages such as cheap, durable, drapable, pliable, and light weight [235,240]. In addition, these can be incorporated into textiles in various forms with excellent washability compared to the previously used solid-state gas sensors [241]. Furthermore, as different gases, volatile organic compounds (VOCs), and humidity can be detected with GO-based gas sensors, these sensors are found to be responsive for detecting any environmental change including the identification of different hazardous materials such as toxic gases, organic vapours, and chemical warfare agents [237,242]. Thus, graphene/polymer composites facilitate keeping the environment safe from various harmful materials [242,243]. Apart from this, human body temperature, blood pressure, and heartbeat can be easily measured with the help of these sensors used as wearable devices. Changes in the conductivity of graphene caused by human body motion or environmental changes, provide the desired results [244]. The fabrication and properties of graphene-based materials widely used in wearable technology are tabulated in Table 4.

### 3.3. Agriculture and Wastewater Management

**Figure 14:**
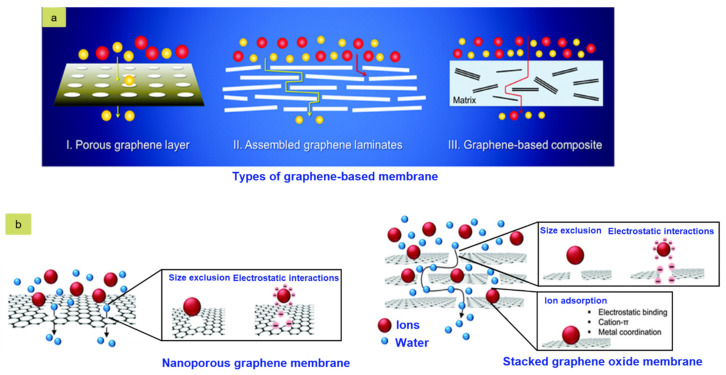
Schematic diagram of (**a**) Different types of graphene-based membranes, (**b**) Water separation membrane; Reprinted with permission [244].

Water contamination with various toxic materials originated from both organic and inorganic sources, for example, industry, agriculture, and household affairs is an alarming topic from the environmental sustainability point of view. Aquatic species are on the verge of extinction due to mass water contamination. Although more than 70% of the Earth’s surface is covered with water, it is becoming increasingly difficult to find clean usable water [249,250]. Therefore, various methods are being used to eliminate water pollution. In the present time, graphene-based nano porous membranes are used as an efficient technique to remove different kinds of pollutants. The nanomembranes effectively act as a barrier for both liquid and gaseous materials. Figure 14 shows a schematic diagram of different types of graphene-based membranes used for wastewater treatment. Table 5 represents the fabrication method and properties of graphene-based materials that are used in agriculture and wastewater management.

### 3.4. Medical and Healthcare

**Figure 15:**
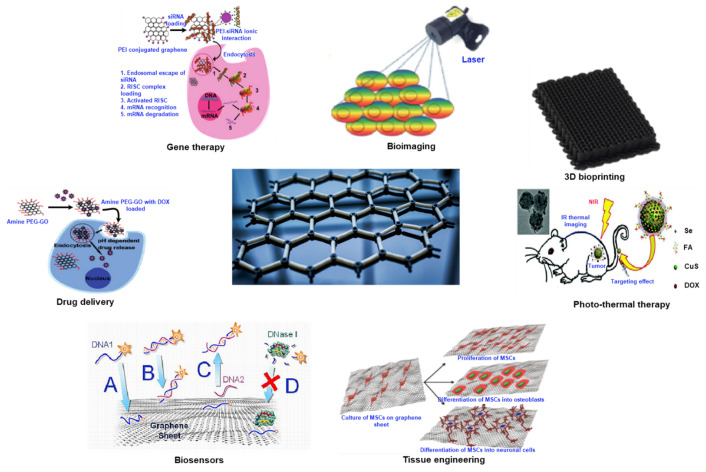
Applications of graphene-based materials in medical and healthcare; Reprinted with permission from [263].

The amazing properties of graphene have laid the foundation of a new horizon of possibilities for various applications and the application of graphene/polymer nanocomposites in the biomedical industry is one of them. Since the first report on the use of graphene in the medical field in 2008 [254,255], much research has been done on its versatility. The large surface area, a strong affinity for hydrophobic drugs, stable chemical properties of graphene, and the enhanced mechanical properties of graphene-based polymer composites made it suitable for a wide range of biomedical applications such as drug delivery, gene therapy, DNA sequencing, tissue engineering, artificial muscles, and cancer therapies as shown in Figure 15.

Because of the atomic thickness and extremely high conductivity properties of graphene, it is extensively used in bioimaging materials. Graphene/regenerated silk fibroins (RSFs) composite fibres produced by the wet spinning method have the potential for being used in tissue engineering, biomedical, and biotechnological areas as they have shown significant antimicrobial efficacy against both gram-positive and gram-negative bacteria [256]. Silica microfibre/graphene oxide can be used for in situ DNA measurement and DNA detection as it exhibits a strong π–π interaction. Moreover, this particular embodiment is sensitive, user-friendly, and can certainly be operated in a hard-to-reach environment [257]. Functionalised graphene/polymer composites are now used as biosensors to diagnose a variety of biomolecules like haemoglobin, glucose, cholesterol, DNA, and even in food industry because of their higher sensitivity towards the changing environments [258,259]. The excellent physical and chemical properties of graphene such as greater surface area, higher absorption ability, higher conductivity, and outstanding catalytic activity made it an excellent choice as a biosensor [259,260]. Although the use of metal-based biosensors were popular due to their electrocatalytic activity, biocompatibility, and lower price, some of these suffered from lower electrical conductivity [260]. Besides, some metallic nanoparticles depicted unreliable signal amplification maybe because of the presence of metallic impurity [261]. Apart from these, it is also an issue of concern that the existence of 50 ppm impurities can cause redox reactions with the biomolecules that ultimately ensures the possibility of toxicological hazards [262]. Similar issues have been found in the case of the biosensors produced with the CNTs and the metallic nanoparticles. Hence, currently, researchers are focusing on the application of graphene-based biosensors to overcome these issues. Table 6 summarises the potential applications of graphene/polymer composites in the biomedical industry. Although graphene or graphene-based composites have received an unprecedented response in the medical sector, more research is needed on the long-term effects of graphene inclusion in the human body.

### 3.5. Automobile, Marine and Aerospace Industry

At present, the emphasis is on application of lightweight but strong materials in various structural engineering industries, especially in the automotive, aerospace, and marine industries [275,276,277,278]. This requirement reduces the parts weight and the amount of fuel required, resulting in considerable reduction in costs and fuel consumption. Less fuel consumption ultimately leads to less carbon dioxide gas emission and environmental pollution. Heavy steel materials are replaced with fibre-reinforced composite materials because of their lightweight and excellent specific strength/stiffness properties. Different types of high strength and high modulus fibre such as glass fibre, carbon fibre, and para-aramid fibre are used as textile reinforcement. In fabricating the fibre-reinforced composites, thermosetting polymer resins are frequently used as the polymer matrix for their chemically inert, thermally stable, and moderate mechanical properties. Although fibre/polymer composites (FPCs) demonstrate substantial enhancement in the in-plane properties, they cannot improve the through-the-thickness properties as desired. In addition, thermosetting polymer resins are very much prone to initiate cracks under cyclic loading conditions. To eradicate these problems, nowadays graphene nanomaterials are incorporated as another reinforcing agent in the FPCs, showing significant improvement both in the in-plane and out-of-plane properties for having a large strength to weight ratio and superior mechanical properties [171,279]. Ford Motor Company has replaced noisy parts with graphene-made parts such as the pumps, fuel rail, chain-driven gears, or belt-driven pulleys on front engines. BMW’s i3 and Volkswagen’s XL1 are examples of commercialised carbon fibre-based polymer composites [280]. Different marine components made of graphene/polymer composites are highly protected from corrosion effect and ultimately demonstrate better service life [281]. At present, different types of ship components like spars, hull, till, rudder, keels, masts, and poles are made from graphene/carbon fibre/polymer composites [281,282]. Graphene materials with their amazing structural strength and conductivity properties have emerged as the prospective contender for numerous applications in the aerospace industry. Graphene/carbon fibre/polymer composites with their super interlaminar shear strength and fracture toughness properties are now used in different parts such as aircraft ribs, panels, fuselages, wings, fuel tanks, and tail assemblies [282]. Around 50% of parts of the Boeing and Airbus aircraft are made of multiscale composites with improved mechanical strength, damage tolerance, thermal stability, and corrosion resistivity properties. Moreover, fibre-reinforced multiscale composites are used considerably in making different components for helicopters such as rotor blades, fan blades, propellers, seats, and interiors [281]. However, despite the fibre-reinforced multiscale composites demonstrate improved properties, the final cost restricted their applications, and more research is required to reduce the costs without sacrificing quality.

### 3.6. Others

Lightweight, strong, and cost-efficient sports items are made from fibre-reinforced polymer composite materials with multi-functional properties. Nowadays, a wide variety of sports items are made of graphene/polymer nanocomposites among them tennis rackets, helmets, hockey sticks, bicycle frames, skis, and golf clubs [281,283,284]. Another promising application area of graphene-based composites is the military defence industry. A wide range of products made from graphene/polymer composites is used in this sector. Because of the higher strength to weight ratio, high stiffness, and other multifunctional properties, graphene-based FPCs are used to make ballistic body armour, drones, and some military automotive parts [285].

## 4. Conclusions and Future Prospect

In this review, the fabrication methods of graphene-based materials and their potential applications in several fields were discussed. The wet spinning process is one of the most followed methods in the fabrication of pure graphene and composite graphene fibres along with natural and synthetic polymers. These fibres are later reduced with chemical or thermal approaches to make electrically conductive graphene fibres. On the other hand, the composite graphene materials incorporating fibres or thermoplastic and thermosetting polymers are fabricated using the matrix modification process, fibre modification process, or the combination of these two processes. These are the cheap, environmentally friendly, and economically feasible approaches to facilitate the industrial large-scale production of graphene-based composite materials. The fabricated graphene materials and their composites are applied to diverse application areas due to the excellent mechanical properties and functional characteristics of graphene and its derivatives. Sensors, nanocomposites, electrodes for solar cells, medical equipment, different sports items, parts, and frames of automobiles are some examples of the application of graphene-based materials. In addition, these materials are also used in wastewater treatment and water purification systems.

Further applications of graphene-based material are expected in nanocomposite industries. Although graphene is currently used in this industry, an excellent understanding of this material is yet to be discovered. Graphene is used in the biomedical and drug-delivery systems; however, focused research is required in the utilisation of all the positive qualities of this remarkable material. This includes investigating the antimicrobial aspects, enhancing biocompatibility, and applying graphene materials to the removal of toxic elements to protect the environment. Apart from these, further research can be carried out to find an optimum and eco-friendly reduction process of graphene that can demonstrate higher electrical conductivity with excellent mechanical properties, which can bring a breakthrough in all application fields. In a nutshell, graphene is a wondrous material in this world that can be tailored in various ways to use in diversified application areas.

## Figures and Tables

**Table 1:** Number of articles published between 2001 and 2020 on the graphene-based composite materials (Scopus, as of 10 September 2021).

Keywords Used for Search	Year Range	No. of Publications
“Graphene based fibres”	2001–2010	57
	2011–2020	3264
“Graphene based fabrics”	2001–2010	0
	2011–2020	410
“Graphene based films”	2001–2010	243
	2011–2020	8658
“Graphene polymer composites”	2001–2010	152
	2011–2020	7655
“Graphene fibre polymer composites”	2001–2010	32
	2011–2020	897

**Table 2:** Number of articles published between 2001 and 2020 on different production methods of graphene-based materials (Scopus, as of 10 September 2021).

Keywords Used for Search	Year Range	No. of Publications
“Wet spinning graphene fibres”	2001–2010	0
	2011–2020	209
“Graphene solution mixing”	2001–2010	13
	2011–2020	719
“Graphene melt blending”	2001–2010	3
	2011–2020	319
“Graphene in situ polymerization”	2001–2010	25
	2011–2020	1817
“Graphene roll to roll milling”	2001–2010	0
	2011–2020	34
“Graphene matrix modification method”	2001–2010	7
	2011–2020	340
“Graphene electrophoretic deposition”	2001–2010	13
	2011–2020	556
“Graphene chemical vapour deposition”	2001–2010	385
	2011–2020	7051
“Graphene chemical grafting”	2001–2010	29
	2011–2020	1341

**Table 3:** Fabrication and properties of graphene-based materials used energy storage and energy conversion devices.

Sample	Fabrication Method	Properties	Ref.
Nanostructured polyaniline (PANi) composited with graphene sheets (GNS)	Chemical polymerisation	The capability of delivering a specific capacitance of 532.3 to 304.9 F/g at scan rates of 2 to 50 mV/s. At a scan rate of 50 mV/s, the exceptionally stable capacitance retention as high as ~99.6%.	[207]
Composite films of chemically converted graphene (CCG) and polyaniline nanofibres (PANI-NFs)	Solution mixing	Mechanically stable with high flexibility, easily bent into large angles and/or shaped into various desired structures. The conductivity of the 44% CCG composite film 10 times higher than that of a PANI-NF film. At a discharge rate of 0.3 A g^−1^, developed supercapacitor devices exhibited a high electrochemical capacitance of 210 F g^−1^.	[208]
Co/Zn–S polyhedron homogeneously embedded between the reduced graphene oxide (rGO) sheets film as a binder-free electrode	In-situ polymerisation	In a 6 M KOH alkaline aqueous electrolyte, a high capacitance of 1640 F g^−1^ was measured at a current density of 1 A g^−1^. When used as the positive electrode alongside activated carbon as the negative electrode to devise an asymmetric supercapacitor (ASC), the developed ASC shows an ultra-high energy density of 91.8 W h kg^−1^ with the power density of 800 W h kg^−1^, The developed device exhibits 90.3% capacity retention after 8000 cycles.	[216]
Tin-graphene tubes	Chemical vapour deposition (CVD)	The developed system has high volumetric and gravimetric capacities, as well as high-rate efficiency and long cycling life. Pairing the Tin-graphene tubes with a commercial cathode material LiNi_0.6_Mn_0.2_Co_0.2_O_2_, the gravimetric and volumetric energy density of complete cells is 590 W h kg^−1^ and 1252 W h L^−1^, respectively.	[217]
Polyaniline hybridised three-dimensional (3D) graphene	Electrophoretic deposition (EPD)	Interacts with bacterial biofilm in three dimensions. Allows easier electron transfer. Achievable multiplexed and high-conductivity pathways.	[215]
Graphene/TiO_2_ continuous fibres (GTF)	Solution spinning and annealing method	Photocatalytic activities under both UV irradiation and visible light irradiation. Under UV irradiation superior activity than the benchmark photocatalyst P25. Four times more effective than pure TiO_2_ fibres (PTF) under visible light irradiation.	[218]
Amino graphene oxide/dopamine modified aramid fibres	Chemical grafting	Exhibits 34% higher interfacial shear strength (IFSS) (35.21 MPa) compared to pure aramid Fibres (AF)/epoxy composites even at a high reaction temperature of 60 °C.	[219]

**Table 4:** Fabrication and properties of graphene-based materials used in wearable technology.

Material	Fabrication Method	Properties	Ref
Graphene/PEDOT (GF@PEDOT) fibre	In situ polymerisation	Specific capacitance of up to 15.39 mF cm^−2^ (0.58 mF cm^−1^) at 0.53 mA cm^−2^.	[220]
Cellulose nanofibres (CNF) reinforced graphene/polypyrrole microfibre	Wet spinning	Superior energy density, remarkable rate capability, and good cycling ability with tensile strength of 364.3 MPa.	[222]
Transition metal oxide nanorods/reduced graphene oxide (rGO) hybrid fibres	Wet spinning	Can be cycled reversibly at a high voltage of 1.6 V. Delivers a superior volumetric energy density. Excellent flexibility, cycling ability, and mechanical stability.	[223]
Cotton/graphene/polyaniline (PANI)	Dip-coating	The energy density of 9.7 µWh cm^−2^. The power density of 840.9 µW cm^−2^. Under the mechanical bending and stretching conditions shows stable electrochemical performance.	[224]
Polyaniline/graphene (PANI/GF) hybrid fibres	Wet spinning	Composite fibres have improved Structural uniformity and stability. High specific capacitance (87.8 mF cm^−2^) and high energy density (12.2 μWh cm^−2^). Current density of 0.22 mA cm^−2^.	[225]
Graphene Fibre with a titanium core (AD:Ti@RGO)	Alternately dipping (AD) method	Ultra-high specific capacitance (up to 1722.1 mF cm^−2^). The highest specific capacitance reported to date.	[226]
Porous polyaniline nanorods/graphene fibres (GF@PANI)	Chemical polymerization	The capacitance of 357.1 mF cm^−2^. Energy density of 7.93 μWh cm^−2^ (5.7 mWh cm^−3^). Power density of 0.23 mW cm^−2^ (167.7 mW cm^−3^). 3.8% capacitance loss after 5000 cycles. Rate capability (78.9% capacitance retention).	[227]
Polyaniline/carbon nanotube graphene fibre	Electrophoretic deposition (EPD)	Spring-like coiled fibre coated with an elastic polymer. Extreme stretchability. Cycles with up to 500% strain for a thousand cycles. The specific capacitance of ≈138 F g^−1^.	[230]
Polyaniline nanorod arrays/graphene (PNA/G)	Chemical polymerisation	Large capacitance (230 mF cm^−2^), High cycling stability (86.9% retention after 8000 cycles), Long-term bending durability and high energy density (37.2 μWh cm^−2^). High electrical conductivity (18,734 S m^−1^). Pseudo-capacitance.	[231]
Sulphur-doped graphene fibres (S-GFs)	In situ Polymerisation	The high specific capacitance of 4.55 mF cm^−2^. The current density of 25.47 μA cm^−2^.	[232]
Graphene/poly (vinyl alcohol) composites as the conductive sheath, and polyurethane yarn as the elastic core	Dip-coating	Two sensors have been developed (graphene concentration of (a) 0.8 wt% and (b) 1.0 wt%, and (a) 12 and (b) 9 cycles of coating, respectively). The change in relative resistance and the applied strain maintains a good linear relationship (correlation coefficient of (a) 0.95 and (b) 0.97). Good repeatability (repeatability error of (a) 2.03% and (b) 1.81%). Hysteresis error of (a) 7.03% and (b) 9.08% implies low hysteresis. Thermal stability is excellent.	[233]
Graphene quartz fibre (GQF)	Chemical vapour deposition (CVD)	Capable to be knitted into meter-scaled knit fabrics. Tunable conductivity sheet resistances of 0.2−10 kΩ/sq). Electrothermal conversion efficiency is up to 980 °C within a few seconds at 24 V. To organic solvent vapour, it has a high sensitivity, a quick response time (<0.5 s), and a long life (~5000 cycles).	[234]
Chitosangraphene oxide composites polymer modified glassy carbon electrode (CS/GO-IIP)	Dip-coating	Under optimised conditions, a linear dependency of 0.5 to 100 µmol/L, with a detection limit of 0.15 µmol/L. Acceptable recovery rates for tap and river water samples.	[239]
Zinc oxide (ZnO) and reduced graphene oxide (rGO) coated wearable cotton fabrics	Coating (In-situ sol-gel method)	The as prepared rGO/ZnO coated cotton (ZnO + 7 wt% rGO) achieves the highest total EMI shielding effectiveness of ~99.999% (54.7 dB). An effective absorption efficiency of 99.99% and capable of shielding impinging EM waves greater than 99.999%.	[246]
Poly (styrene-butadiene-styrene) (SBS)/graphene (Gr) composite fibre-based flexible strain sensor	Wet spinning	The fibres with 5 wt% graphene have a wide response range of up to 100% strain. The SBS-5 percent Gr composite fibres have excellent sensing efficiency when it comes to detecting human upper limb movements at various joints.	[247]
graphene (G), carbon black (CB), and polydimethylsiloxane (PDMS) into three-dimensional (3D) Ni sponge	Dip-coating	The G/CB/Ni strain sensor is flexible, has a high sensitivity (gauge factor of 138 at 16% strain), and is stable over time. The G/CB/Ni sensor can accurately track subtle human movements like pulsing, blinking, and swallowing, as well as can measure the strength of muscles. The G/CB/Ni sensor can be used to detect human movements in humid and wet environments since it is waterproof.	[248]

**Table 5:** Fabrication and properties of the graphene-based materials used in agriculture and wastewater management.

Material	Fabrication Method	Properties	Ref.
Graphene oxide/iron (GO-Fe) composite	Solution mixing	Phosphate ions are attached to the GO-Fe composite, resulting in a loading capacity of 48 mg P/g. Compared to commercial mono ammonium phosphate (MAP) fertiliser, a GO-Fe composite loaded with phosphate (GO-Fe-P) fertiliser resulted in a slower release of P, minimizing the risk of soluble P leaching or runoff into surface and ground waters.	[251]
Water-soluble graphene	Modified Hummers and Offeman’s process	After 20 days of exposure, graphene significantly decreased the growth of plant and biomass under experimental conditions as compared to a control. The number and size of leaves on graphene-treated plants reduced in a dose-dependent manner. Under the same conditions, lettuce seedlings showed little or no substantial toxicity.	[252]
Graphene quantum dots (GQDs)	Chemical grafting	The growth rate of leaves, roots, shoots, flowers, and fruits accelerated by graphene quantum dots.	[253]

**Table 6:** The potential applications of graphene/polymer composites in the biomedical industry.

Applications	Purpose	Graphene/Polymer Composites	Ref.
Neuroscience	Neural sensing and stimulation	Porous graphene microelectrode array	[264]
Gene delivery	Drugs and genes delivery	PAMAM-GO	[265]
	Cancer therapy	GQD-PEI-Dox-GFP (GIDG) GQD-PEI-EGFR-Dox (GIED)	[266]
	Optimisation of the gene delivery system	GO-APTES	[267]
Biosensors	Detection of DNA	PANI/GP	[268]
	l-lysine biosensing	c-MWCNTs–SnO_2_–GR–CS	[269]
	Glucose sensing	3D NiO hollow sphere/rGO composite modified electrode	[260]
	Amperometric uric acid detection	Uricase/Chi-Gr cry/PB/SPCE	[270]
Drug delivery	Controlled release of the Sumatriptan Succinate (SS) drug	CS-TPP-GO	[271]
	Cisplatin drug loading efficacy	CS/M/S/GO	[272]
	Specificity to tumour cells	MGO-MIP	[273]
	Therapeutic efficacy of doxorubicin (DOX) as an anticancer drug	PB-MG	[274]

PAMAM = polyamidoamine; PANI = polyaniline, GP = graphene, c-MWCNTs = carboxylated multiwalled carbon nanotubes; CS = Graphene–chitosan; Cry = cryogel; Gr chi = graphene-incorporated chitosan; PB = Prussian blue; SPCE = screen-printed carbon electrode; CS-TPP-GO = Chitosan/tripolyphosphate/Graphene oxide hydrogel; CS/M/S/GO = chitosan-coated magnetite, silicon dioxide, and graphene oxide; MGO = magnetic GO; MIP = Molecularly imprinted polymers; PB = brush polymer; MG = magnetic graphene oxide; GQD = graphene quantum dots; PEI = polyethylenimine; Dox = GFP = green fluorescent protein; Dox = drug doxorubicin; EGFR = epidermal growth factor receptor; APTES = 3-aminopropyltriethoxysilane.

## Data Availability

Not applicable.
